# Verbal consent in biomedical research: moving toward a future standard practice?

**DOI:** 10.3389/fgene.2025.1472655

**Published:** 2025-02-28

**Authors:** Alycia Noë, Emilie Vaillancourt, Ma’n H. Zawati

**Affiliations:** Centre of Genomics and Policy, Faculty of Medicine and Health Science, Victor Philip Dahdaleh Institure of Genomic Medicine, McGill University, Montreal, QC, Canada

**Keywords:** consent, verbal consent, biomedical research, COVID-19, rare disease

## Abstract

Properly obtaining informed consent is a core obligation for research conducted using human subjects. The traditional informed consent process involves written forms and obtaining signatures. This process remains the standard, but in various research settings, such as COVID-19 and rare disease research, verbal consent has increasingly become the norm. Although verbal consent is used in these settings, its use is still a subject of debate. This article reviews in what medical settings verbal consent is commonly seen today, various advantages and disadvantages of verbal consent, and its legislative and policy ecosystem. In doing so, this review article asserts that it is time for the debate over verbal consent to come to an end and for legislator and policymakers to acknowledge its use and to formalize the process. This will allow verbal consent to be regulated in a similar manner to written consent and will give clinician-researchers guidance on how to better implement verbal consent in their studies to addressing ongoing concerns with the consenting process as a whole.

## 1 Introduction

Traditionally, informed consent for research purposes has been obtained using written consent forms as these forms were viewed as the quickest and most-cost effective way to document consent (Broekstra et al., 2017, p. 195). These consent forms were presented to patients by their physician, accompanied by a conversation prior to signature. While written consent remains the standard practice in medical settings (Thompson and MacNamee, 2017, p. 51), informed consent has increasingly been obtained using alternative consenting models, such as electronic consent forms and verbal consent. These new models of consent present an opportunity to improve the consenting process and stray away from the classical belief that signing a written form is to only way to protect patients and research participants.

These alternative models became increasingly relevant during the COVID-19 pandemic, where standard research practices needed to be rapidly adapted to conform with public health measures. For example, Health Canada exceptionally allowed informed consent for clinical trials to be obtained using alternative methods, such as video-teleconferencing (Health Canada, 2023). These developments, required during COVID-19 to keep ongoing clinical trials running, raised questions as to whether seemingly temporary alternative informed consent models will become the new norm in our post-pandemic world. This article will focus on the adoption of verbal consent in biomedical research and clinical practice. Specifically, it will evaluate how verbal consent has been adopted by researchers and why its use has been favoured in certain research contexts. This is a necessary investigation as the use of verbal consent in biomedical research has been explored only sparsely in the literature and it is unclear what the status of verbal consent is post-pandemic.

This article will begin by discussing the characteristics of verbal consent in biomedical research and medical care. Then, the use of verbal consent will be evaluated in three sample cases–COVID-19 research, rare disease research, and clinical visits. Finally, we assess the future for verbal consent in clinical care and research and the legal basis for its use. Specifically, we evaluate the advantages and disadvantages of the use of verbal consent and highlight the fact that verbal consent does not alone address the need for a more robust consenting process in medical research and clinical settings.

In exploring these topics, we hope this article fosters further discussions about verbal consent as a standard approach to informed consent in research, both in Canada and internationally. We also hope that this article draws attention to the potential pitfalls of verbal consent, which must be addressed in its continued adoption to ensure an evolution in informed consent that addresses issues in medical consent, rather than continues to ignore them. We highlight that it is important to keep aware that while some elements of verbal consent have worked well, there remain aspects of the consenting process that need further consideration to allow verbal consent to work better in the future.

## 2 Verbal consent in biomedical research and medical care–characteristics and communication

### 2.1 What is verbal consent?

In general, verbal consent varies from written consent, in that it is obtained verbally rather than in written form. In practice, this means that no consent form is signed, rather research participants and patients are provided with the information necessary for proper consent verbally and then, once informed, consent verbally (Kakar et al., 2014, p. 69). There are numerous methods to obtain verbal consent. It can be obtained remotely, via telephone or videoconferencing, and in-person (Garg and Khanna, 2021, p.11; Skelton et al., 2020, p. 1). Nonetheless, the physician or researcher that obtains the consent must still make note of the fact that consent was obtained (e.g., in the patient’s file). Therefore, there remains a level of necessary documentation in verbal consent, but the documentation does not implicate the patient or participant themselves.

Overall, verbal consent is appealing as it makes the informed consent process a more natural conversation than when a written form is used. The conversation can be ongoing, which is considered to be the hallmark of proper informed consent processes (Canadian Institutes of Health Research et al., 2022). However, they are also worries with the rapid adoption of verbal consent in biomedical research contexts, such as poor understanding and retention of the risks and benefits to the participants themselves (Nusbaum et al., 2017, p. 1074).

### 2.2 Verbal consent in biomedical research

In Canada, verbal consent for research-related activities is acknowledged as an ethically equivalent alternative to traditional written consent (Canadian Institutes of Health Research et al., 2022). When there are valid reasons for using verbal consent, the process used to attain consent must be adequately documented (Canadian Institutes of Health Research et al., 2022). Adequate documentation includes, for example, a copy of the consent script used or a written summary of the information provided to the participant, and a clear description of how verbal consent was obtained (e.g., detailed notes or audio recording) (Children’s Hospital of Philadelphia, 2022). This documentation is important for any issues or disputes that arise from the consenting process and provides safeguards for research participants.

However, even with the requirement of documentation, there still exist regimes under which signed written consent forms are mandatory and seemingly cannot be substituted with verbal consent (Govt of Canada, 1986). Importantly, the requirements for mandatory signed consent forms are often found in legal statutes (i.e., hard law), whereas the acknowledgements of verbal consent are often found in policy instruments (i.e., soft law). To overcome this barrier,clinician-researchers are always looking for ways to ensure the validity of the informed consent of participants (Baer et al., 2011, pp. 124–125). For example, the verbal consent processes in research settings often include audiovisual tools to enhance the participants understanding and retention of the information presented to them during consent conversations (Knoppers et al., 2020, p. 5). In the same line, verbal consent in research settings often incorporates elements of electronic consent (e-consent), whereby patients are informed using digital means, such as phones, tablets, or computers without any live interaction with research staff (Shenoy, 2015, p. 173).

Many research ethics boards (REBs) across Canada have issued guidelines and templates for verbal consent since the beginning of the COVID-19 pandemic. These templates are meant to provide examples of the proper approach to verbal consent to ensure the rights of research participants are safeguarded and to avoid inconsistencies in the consenting procedure. Examples of REBs that have done so include, but are not limited to, SickKids, Ottawa Health Science Network, and the University of Calgary (SickKids, 2022; Ottawa Health Science Network Research Ethics Board, 2022; University of Calgary, 2014). Generally, REBs permit the use of verbal consent where they are satisfied that the research is of minimal risk to participants and it is impractical to carry out the research without verbal consent (Canadian Institutes of Health Research et al., 2022). A common approach to regulation of verbal consent by REBs is a requirement of submission of the verbal consent script for review and approval by the REB before it can be used with research participants (Ottawa Hospital Research Institute, 2020). Many also require a paper copy of the script to be sent to participants in advance of the conversation (University of Alberta, 2020).

## 3 Verbal consent in use cases

### 3.1 COVID-19/viral sampling

As previously mentioned, research performed during the COVID-19 pandemic required the adoption of alternative informed consent models, such as verbal consent. This shift is best illustrated by research involving participants with COVID-19 itself, as this research was time sensitive as it helped shaped the public health responses to the virus and required adaptation of typical informed consent guidelines.

Obtaining written informed consent from these patients proved difficult due to the initial uncertainty of the infectious nature of the virus and the fact that many patients were far too ill to properly complete the formalities required for written consent (Knoppers et al., 2020, p. 4). Methods of obtaining informed consent from these patients needed to prioritize limiting exposure to the virus (Garg and Khanna, 2021, p.11). Furthermore, at the height of the pandemic, in-person interactions were further restricted due to shortages of personal protective equipment (PPE) (Garg and Khanna, 2021, p.11). The solution to address these concerns was verbal consent. The rapid acceptance of verbal consent as the default method of consent during COVID-19 was largely due to their being little to no other alternative methods to obtain informed consent.

In the context of biobanking of viral samples, clinician-researchers leaned into tele-consenting, whereby patients consent verbally over the phone or videoconference after being informed verbally of pertinent information (Allocca et al., 2020, p. 543). Completing the consenting process while physically distanced allowed as little exposure to the virus as possible. Upon consent, nurses or clinicians protected by PPE could then collect samples (e.g., respiratory secretions, blood samples) as quickly as possible (Knoppers et al., 2020, p. 4). It is also important to note that while verbal consent was essential for COVID-19 research, there were also difficulties with its adoption due to lack of legal and policy guidance. For example, the script for verbal consenting needed to be validated and adopted the REB, however, the script was not necessarily followed in detail by all undertaking consenting processes. This led to varying degrees of consent amongst participants–an issue that must not be ignored in the adoption of verbal consent in biomedical research today.

In gist, verbal consent and tele-conferencing technologies enabled research into COVID-19 using human participants to occur during the pandemic. This research was important to shape immediate public health responses, to develop a vaccination, and to begin to understand the long-term consequences of COVID-19 infection.

### 3.2 Rare diseases research

Informed consent for rare disease research is often complex, as consent during the COVID-19 pandemic was but for different reasons (Nguyen et al., 2019, p. 2). The complexity of informed consent in rare disease contexts has been increasing due to technological and genomic advancements, the necessity of pooling of data due to the small number of patients with these diseases, and the recruitment of children for these studies (Nguyen et al., 2019, p. 2). For example, certain rare disease research may require the collection of additional data when compared to research into other conditions. While it has come to be expected that general indicators of health like blood test results may be stored during research, certain rare diseases may require the collection of additional data such as audiovisual data from videos or facial imaging (Nguyen et al., 2019, p. 2). The increased diversity of data collected in rare disease research can pose a unique privacy concern that is further compounded by the necessity of data sharing due to the scarcity of patients and the use of children for much of this research. Due to these complexities, the consent forms for rare disease research can be highly technical and lengthy, perhaps rendering them as not an ideal approach to obtain informed consent (Nguyen et al., 2019, p. 3).

Furthermore, patients or parents of patients with rare diseases tend to be already well informed on the disease and treatment options, making verbal consent a more ideal consent model in rare disease research than in other research settings (Budych et al., 2012, p. 155). In fact, verbal consent has already been implemented in various rare disease studies, such as the Rare United Kingdom Diseases (RUDY) Study platform, where patients consent over the telephone after research personnel explain the study to the patients using a prepared script (Javaid et al., 2016, p. 3).

## 4 The future of verbal consent and the basis for its use

### 4.1 The patchy Canadian legislative framework for verbal consent

As shown above, verbal consent is used in specific situations in Canadian biomedical research today, however, there remains a debate as to the future of verbal consent in clinical and research settings and the legal basis for its continued use. The ethical and legal framework governing biomedical research in Canada allows for the use of verbal consent under certain conditions. However, the use of verbal consent has largely been imagined as a temporary measure to ensure the continuance of research in exceptional circumstances.

While federal legislation does not acknowledge the possibility of verbal consent, there are many federal guidance documents that do, such as the *Tri-Council Policy Statement: Ethical Conduct for Research Involving Humans (2022)* (TCPS2) (Canadian Institutes of Health Research et al., 2022). Many provinces also explicitly provide for verbal consent in their healthcare consent legislation. This is seen in article 24 of the Civil Code of Québec (*Civil Code of Québec*, 1991), section 9(1) of British Columbia’s *Healthcare (Consent) and Care Facility (Admission) Act* (*Healthcare (Consent) and Care Facility (Admission) Act*, 1996), and section 5(2) of Ontario’s *Healthcare Consent Act* (*Healthcare Consent Act*, *1996)*. However, Manitoba and Nova Scotia are among the provinces that still have legislative provisions that mandate written consent (The Health Care Directives Act, 2008). The provinces that still mandate written consent and have no provision allowing for other forms of consent are interesting as many guidance documents from hospitals in these regions explicitly allow for verbal and other forms of consent, in addition to written consent (IWK Health Centre, 2019; Alberta Health Services, 2010). While this seems to be blatantly in conflict with the consent legislation, there is no jurisprudence that challenges the practice of the ongoing use of verbal consent in these regions.

There is a complication for clinical trials that wish to use verbal consent. The Canadian *Food and Drug Regulations* still necessitate the use of written informed consent. This is underscored in section C.05.010(h) (Food and Drugs Regulations, 1985), which necessitates that every sponsor of a clinical trial shall ensure that “written informed consent […] is obtained from every person before that person participates in the clinical trial”. Both the TCPS2 and the Govt of Canada 2023 *for Part C, Division 5 of the Food and Drugs Regulations* reiterate the necessity for written consent forms for clinical trials involving human subjects (Food and Drugs Regulations, 1985). Furthermore, the guidance document details that the *Food and Drugs Regulations* should be interpreted in accordance with the International Council for Harmonisation (ICH) guidance–*Integrated Addendum to E6(R1): Guideline for Good Clinical Practice E6(R2)*, which, yet again, requires the use of a written consent form (International Council for Harmonisation of Technical Requirements for Pharmaceuticals for Human Use, 2016).

As federal guidance documents and provincial legislation both allow for the use of verbal consent, perhaps federal legislation, such as the *Food and Drugs Regulations*, should be adapted to outline circumstances in which signed consent forms can be waived (?), although this may also be too restrictive of an approach. This is especially important considering that primary guideline for research ethics that is furnished by the Canadian government, the TCPS2, explicitly states the benefits of using verbal consent when circumstances necessitate it (Canadian Institutes of Health Research et al., 2022).

### 4.2 Accessibility and equity in research

One of the circumstances outlined by the TCPS2 as worthy of using verbal consent is when cultural norms require it (Canadian Institutes of Health Research et al., 2022). The guidelines state:

“In some types of research, and for some groups of individuals, written signed consent may be perceived as an attempt to legalize or formalize the consent process and therefore may be interpreted by the participant as a lack of trust on the part of the researcher. In these cases, oral consent, a verbal agreement or a handshake may be required, rather than signing a consent form.”

An acknowledgement that the long-established method of obtaining informed consent, a signed paper form, may need to be set aside for the purposes of more robust informed consent is in line with the principles of consent, namely, supporting autonomous decision making and the building of trust between the patient and their physician (Hall et al., 2012, p. 533; Eyal, 2014, p. 437). Verbal consent will also allow for broader inclusion of historically under-represented groups in research, an important aspect to realize article 12, the right “to the enjoyment of the highest attainable standard of physical and mental health”, of the *International Covenant on Economic, Social and Cultural Rights* (*ICESCR*) (International Covenant on Economic, Social, and Cultural Rights, 1966).

This access is largely due to the ability of verbal consent processes to help overcome the distrust of the healthcare system felt by some groups of individuals (Hughson et al., 2016, p. 3). For example, Indigenous communities in Canada and around the world have been excluded from research, namely, due to distrust of the medical field leading to reluctance to sign written consent forms that may not be well-understood (Wilson, 2008, pp. 15–17). Verbal consent, as it feels much more like a conversation, can help to build trust with communities who have been historically mistreated by the medical profession (Cumyn et al., 2020, p. 6).

Furthermore, written informed consent, more so than verbal consent, inherently excludes those based on linguistic literacy, health literacy, and comprehension levels (Raimondo et al., 2014, p. 5; O’Sullivan et al., 2020). Spoken words are easier to understand who those who may not have had the opportunities to learn to read complicated texts (Benitez et al., 2002, p. 1407). Although the proper consent process should always involve a conversation in conjunction with the paper consent form, occasionally, the mere introduction of a paper form derails the trust built through these conversations. This occurs for numerous reasons. Firstly, as the TCPS2 implies, some cultural groups favour verbal information over written information, as the spoken word is viewed as the pinnacle of reliability (Hughson et al., 2016, p. 3, 6; Castro et al., 2015, p. 511). Furthermore, written forms are viewed as formal or legal documents by many patient groups and are met with hesitation (Canadian Institutes of Health Research et al., 2022).

Importantly, in the Canadian context, verbal consent provides an opportunity to overcome systemic barriers to research and healthcare for Indigenous communities. Indigenous communities also have experienced a deep history of paper documents being manipulated to their detriment. For example, many paper copies of treaties were signed with an “X” next to the Chief’s name, rather than a true signature. These signatures also occurred after very little discussion of what the paper text said (Cook et al., 2021, p. 141). Indigenous peoples were asked to trust that the paper document reflected their wishes and interests (Vallance, 2015, p. 26). To make matters worse, new clauses were added to many of these treaties, without the consent of the Indigenous communities concerned, after the treaties were signed (Albers, 2017). With a history like this, Indigenous peoples today remain cautious of paper forms and rightfully so. The history of paper documents being used to hide true intentions shows the fallacy behind the belief that written forms are the only way to truly protect an individual’s interests.

Furthermore, as many Indigenous peoples communicate primarily using verbal communication, the use of verbal consent can indicate a sign of respect and deference towards their cultural norms, rather than forcing paper forms. This a similar idea to how the communication of forestry projects on Indigenous territory has been reimagined. Instead of using difficult to engage with technical information (e.g., maps and reports), urban planners have begun to explore the use of realistic 3D visualisations of the future landscape after forestry projects (Lewis and Sheppard, 2006, p. 291). This allows a greater level of shared understanding between those pitching a forestry project and the communities that will be most greatly affected by that project. The use of verbal consent permits the same to occur in research and healthcare. For example, researchers who identify as Indigenous have described adopting storytelling approach to informed consent (Wilson, 2008, pp. 8, 126), whereby only oral consent is used to reduce the power imbalances between researchers and Indigenous patients (Fitzpatrick et al., 2016, p. 4).

Additionally, the adoption of verbal consent can allow for greater access to healthcare and research studies for marginalized communities that may be fearful of their identity being disclosed by participation. This can include individuals with stigmatized identities or those engaging in illicit activities (e.g., gay men living with HIV, transgender women engaged in sex work, individuals with drug addictions) (Abrams et al., 2020, p. 6). Just as with Indigenous communities, the thought of signing a physical document where the individual must record their name. Although verbal consent still needs to be documented in some way, even if simply by recording it in the participant’s file, it is often viewed as more respectful of the participant’s privacy (Abrams et al., 2020, p. 6). Verbal consent represents an important step to address the power dynamics inherent to consenting in healthcare contexts.

Similarly, verbal consent may help with fears that surround being considered a test subject or fears of receiving new treatments. Verbal consent can help to build a better rapport than written consent, although this remains to be tested in the literature in a quantitative way.

While verbal consent offers many possibilities of improving equity and inclusion in medical research, it also presents some pitfalls, many of which are the same pitfalls that accompany written consent. For example, verbal consent does not help with barrier to participation in clinical research such as psychological issues (e.g., denial or depression) and financial burden (e.g., whether the treatment received will be covered by insurance) (Nijhawan et al., 2013, p. 138). These are issues often faced by already marginalized populations. These will need to be addressed for verbal consent to reach its full potential of making research a more inclusive environment.

Overall, verbal consent offers an opportunity for researchers to remove linguistic and cultural barriers to healthcare services and research. Furthermore, when combined with electronic technologies, verbal consent may help to remove geographic barriers to the participation in research (Maspero et al., 2020, p. 2), as some of the steps for the research project can be done remotely, requiring less travel expenses from the patients (Byrne and Watkinson, 2021, pp. 65).

### 4.3 How strong is the case for the future use of verbal consent?

Verbal consent in medical care and research is happening. Especially since the COVID-19 pandemic, verbal consent has been used more widely in the medical context. Thus, rather than fighting the inevitable, policy and law must continue to acknowledge, rather than ignore, the realities. This has been done in Québec where, as of 2013, the law that required written consent for research was changed to allow for consent in the form of something “otherwise than in writing” (Civil Code of Québec, 1991). Other provinces have followed suit. It is especially important for legal guidelines that discuss clinical trials and for the practice of REBs to adapt with our changing consent practices in medicine.

The normative preferability of written consent over verbal consent in clinical and research contexts is the primary hurdle that stands in the way of a more universal adoption of verbal consent. This includes, but is not limited to, reticence of REBs leadings to a slower ethics approval process, hesitation of researchers due to worries over liability, and concerns over Health Canada legislation concerning consent in clinical trials and drug approval (regardless of the statements in TCPS2 2022) (CMPA, 2023; Lawton et al., 2017, p. 6). Therefore, even if legal and ethical norms continue to develop to permit further use of verbal consent, its adoption as a standard practice by physicians will also depend on researchers’ acceptance of the practice and support of its use by researcher institutions/REBs. The provision of training on verbal consent for researchers from their research institutions would help to aid in this transition.

Other hurdles to the adoption of verbal consent are privacy concerns. First and foremost, since verbal consent occurs verbally, there is a major concern that individuals may consent without fully understanding (Bossert and Strech, 2017). This issue also exists with written consent (Isles, 2013, p.1), but ideally if the norms of consent are changing, they will improve issues with traditional consenting practices.

Much can be done to evaluate whether a patient/participant understands what they are consenting to. Tests and teach-back components can be integrated into verbal consent (Glaser et al., 2020, p. 139). If the tests and teach backs show insufficient understanding, further verbal information can be given to improve understanding prior to consent (Glaser et al., 2020, p. 121). Audiovisual aids can also improve the understanding of individuals (Loftus et al., 2020, p. 4). Furthermore, it remains important with verbal consent for a clinician-researcher to stay aware of indications that a patient is overwhelmed by the information being presented, whether that be due emotional overwhelm or informational overload (Bester et al., 2016, p. 875).

Other privacy concerns include how the documentation of verbal consent is stored. Often, verbal consent is documented with audio or video recordings. This means that further information is stored beyond a signed form that is associated with written consent. While this manuscript is focused on the Canadian landscape, privacy law worldwide is influenced by the General Data Protection Regulation (GDPR) of the European Union. The GDPR sets regulations for how to collect consent and how to keep records of consent. In the case of verbal consent documentation, a patient or participant would need to be (1) informed that their personal data is being collected (i.e., the storage of the verbal consent documentation), (2) have the right to access and correct this data, (3) have the right to data portability and erasure, (4) have the right to restrict the processing of their health data, and (5) have the right to complain. In principle, however, the concerns associated with the storage of verbal consent documentation are no different than the concerns with other health information. This is made clear in publications concerning Canadian health information and the implications of the GDPR (Information and Privacy Commissioner of Ontario, 2018).

Another issue with the adoption of verbal consent in clinical and research settings is the time pressures faced by healthcare professionals (Shah et al., 2024, p.7). It remains a reality that written forms require less time than a verbal consenting procedure. This is the hard to avoid truth of verbal consent and indicates that universal adoption of this type of consenting process will require a shift in the priorities and pressures in our healthcare system. Verbal consent is not perfect, but it can represent a step in the right direction.

## 5 Discussion

Obtaining informed consent from research participants is the cornerstone of modern research ethics. To date, the signing of a written consent form remains the standard of practice. However, informed consent is intended to involve much more than that. The best version of informed consent involves providing participants with information on the research to be performed, helping participants comprehend this information, and answering participants’ questions, both at the time of consenting and in the future.

The COVID-19 pandemic has necessitated a shift away from in-person interactions, further highlighting the existing need to obtain informed consent in forms other than in writing. This article has identified many benefits of the use of verbal consent, such as better inclusion in research and improved understanding from participant, although more research is needed on these areas. It has also highlighted how the use of verbal consent is becoming more and more the norm in clinical and biomedical research settings. Nonetheless, the use of verbal consent in medical spaces still remains under debate. This is due to the fact that verbal consent does not overcome all the issues associated with consent in the healthcare context and to the legal and policy environment surrounding healthcare consent.

While laws and regulations concerning consent in healthcare contexts have begun to change to explicitly allow for verbal consent on a provincial level, federal laws are slow to catch up. There is hope on the federal level, however, as verbal consent was included as a means to address cultural hesitancy to healthcare in the TCPS 2 2022. Furthermore, post-COVID-19 pandemic, verbal consent is being used more than ever, showing continued acceptance of its use by researchers and REBs. If federal legislation adapts and REBs continue to show more acceptance of verbal consent, this offers an opportunity to refine verbal consenting processes to better address concerns (e.g., mechanisms of review for verbal consent processes, evaluations of patient/participant understanding).

Much energy is expended on the dispute as to whether verbal consent is an appropriate consenting process, but this is the wrong question to be focused on. The reality is that verbal consent has been accepted, with open arms, by patients, researchers, and clinicians. Rather than debate the use of verbal consent in circumstances that necessitate flexible thinking around consent, we need to focus on formalizing the verbal consenting process, with supporting guidelines, policies, and laws to provide additional safeguards to patients and research participants. Verbal consent, in cases like COVID-19 and rare disease research, has shown its efficiencies. Therefore, we should aim to make verbal consent accessible, readily understandable, and, without a doubt, innovative. Verbal consent should also continue to be practiced without forgetting to addressing the challenges with the consenting process in healthcare more generally.

Innovation is still needed to navigate certain challenges with verbal consent. Further research and resources are required to familiarize and inform key stakeholders, mainly researchers, research institutions and REBs, on verbal consent. These groups have shown more uncertainty as to whether to adopt verbal consent in their clinical trials and research.

Additionally, there also remain open questions concerning verbal consent that should be explored. For example, there are concerns about the risks of medical paternalism with verbal consent versus written consent and about the risks of abuse due to verbal consent being a less regimented way of consent. These need to be addressed, perhaps with formalization and standardization. Furthermore, while there is preliminary evidence of comparable comprehension levels of verbal consent compared to written consent (Kashur et al., 2023, p. 3), continued research on the topic could lead to greater stakeholder acceptance of verbal consent and wider use. Verbal consent stands to become a readily approachable method of consent. With patients’ attention spans becoming shorter in the age of social media and quick consumption, verbal consent can be adapted to be more informative and more relatable than written consent models. Rather than expecting a patient’s comprehension to rise to the level of a written consent form, as there is evidence that many written consent forms are well-above the recommended reading levels of the general population (Hitchcock et al., 2020, p. 2), verbal consent can easily be adapted to what the patient needs to better understand. Furthermore, as consent is meant to be an ongoing conversation, even when written methods are used, the formalization of verbal consent in healthcare contexts serves to reinforce the conversational ideal for consent. Exploring alternative methods of informed consent is urgent. The golden standard of a signed form is becoming more antiquated as the years pass.

To end, while some argue to verbal consent is shifting the medium of consent, this article highlights that, in practice, it is not. Written consent forms were merely the physical outcome of the informed consenting process. This process, when done correctly, also involved conversations with participants and patients (i.e., the elements of the verbal consenting process). The elimination of the written consent form merely entails the elimination of the final step in the informed consent process. And if the elimination of a physical form that must be signed helps patients and participants feel more trusting and informed during the doctor-patient interaction, perhaps this is a change to lean further into in the future. All this being said, some of the issues seen with written consent persist with verbal consent and must remain top of mind for clinicians and researchers to ensure that verbal consenting processes are adopted in a responsible way.

This article serves as a first step in the bend towards verbal consent. It reflects on the benefits, challenges, and the legal and policy environment that surrounds verbal consent, all of which will be key to its continued adoption and the improvement of the consenting process overall. It also proposes the ratification of verbal consent by incorporating it into more consenting laws and policies.
